# *LRRK2*: Cause, Risk, and Mechanism

**DOI:** 10.3233/JPD-130192

**Published:** 2013

**Authors:** Coro Paisán-Ruiz, Patrick A. Lewis, Andrew B. Singleton

**Affiliations:** a Department of Neurology, Psychiatry, and Genetics and Genomic Sciences, Icahn School of Medicine at Mount Sinai, One Gustave L. Levy Place, NY, USA; b Friedman Brain and Mindich Child Health and Development Institutes, Icahn School of Medicine at Mount Sinai, One Gustave L. Levy Place, NY, USA; c Department of Molecular Neuroscience, UCL Institute of Neurology, University College London, Queen Square, London, UK; d School of Pharmacy, University of Reading, Whiteknights, Reading, UK; e Laboratory of Neurogenetics, National Institute on Aging Intramural Research Program, Bethesda, MD, USA

**Keywords:** LRRK2, associated phenotype, disease risk, biology, future challenges, Parkinson's disease, parkinsonism, genetics

## Abstract

In 2004 it was first shown that mutations in *LRRK2* can cause Parkinson's disease. This initial discovery was quickly followed by the observation that a single particular mutation is a relatively common cause of Parkinson's disease across varied populations. Further genetic investigation has revealed a variety of genetic ties to Parkinson's disease across this gene. These include common alleles with quite broad effects on risk, likely through both alterations at the protein sequence level, and in the context of expression. A great deal of functional characterization of LRRK2 and disease-causing mutations in this protein has occurred over the last 9 years, and considerable progress has been made. Particular attention has been paid to the kinase activity of LRRK2 as a therapeutic target, and while it is no means certain that this is viable target it is likely that this hypothesis will be tested in clinical trials sooner rather than later. We believe that the future goals for LRRK2 research are, while challenging, relatively clear and that the next 10 years of research promises to be perhaps more exciting than the last.

## Introduction

The identification of *LRRK2* mutations as a cause of PD in 2004 had an instant, significant, and lasting impact on our understanding of Parkinson's disease (PD) [1, 2]. This work provided surprising insight into the genetic basis of this disease, revealing that mutations underlie a substantive number of PD cases throughout the World. The protein product of this gene, a kinase, also provided hope, as it was immediately suggested that this would prove to be a druggable target for treating both genetic and idiopathic forms of PD. In this article we will review and discuss the significant progress that has been made in understanding the role of *LRRK2* in PD, both from the perspective of genetics, and through understanding the etiology and pathogenesis of this disorder.

## Parkinson's Disease-Associated *LRRK2* Mutations

**Fig. 1:**
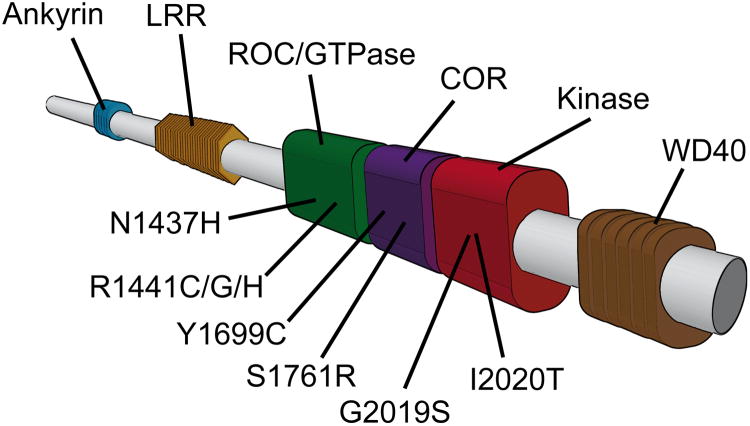
LRRK2 ideogram showing functional domains and penetrant mutations.

Mutations in *LRRK2* (NM_198578.3) are the most common genetic cause of late-onset Parkinson's disease (PD) identified to date [1, 2]. *LRRK2* consists of 51 coding exons and encodes a large 2,527-amino acid protein called dardarin, which consists of several leucine-rich repeats (LRRs), a Ras-like GTPase domain (ROC) along with its C-terminal domain (COR), a kinase domain, as well as a WD40 motif. Although over 100 *LRRK2* mutations have already been reported [3], only a few have been proven to cause PD. These include the p.N1437H, p.R1441C/G/H, p.Y1699C, p.S1761R, p.G2019S, p.I2012T, and p.I2020T mutations [4–7] (Fig. 1). Interestingly, all established pathogenic mutations are clustered among the three domains that form the enzymatic core of dardarin and are associated with variable degrees of population-specificity [7].

The most frequent *LRRK2* mutation, p.G2019S, while barely present in Asia (<0.1%), is responsible for up to 10% of apparently sporadic PD and up to 42% of familial PD [5, 8]; although this mutation has a worldwide distribution, it is present with a higher frequency in Portuguese (16%), Ashkenazi Jewish (28%), and North African Arab (42%) populations [9–11]. Similarly, within the Basque population the *LRRK2* p.R1441G mutation presents at a frequency of 2.5% and 46% in apparently sporadic and familial PD, respectively but is hardly present in other European populations, including other regions of Spain, or North and South America [12–15]. In contrast, the p.R1441C mutation represents the second most common *LRRK2* mutation identified in Europe, being the major genetic cause of PD among Belgian PD patients, likely due to a founder effect [16]. Although the p.R1441G/C mutations have not been reported among Asian PD patients, the p.R1441H mutation has been found in Asia, Europe, and North America [17, 18]. Only four families have been described with the p.I2020T mutation, notably however, this includes the Sagamihara kindred, the first reported family with PD linked to the *LRRK2* locus [19]. Lastly the p.Y1699C mutation, sitting within the COR domain, has been reported in several families of British (1), German-Canadian (1), and Korean (1) origin [1, 2, 20].

While it is likely that at least some of the other reported mutations in LRRK2 are pathogenic, in most instances the segregation or association data are insufficient to prove pathogenicity. Consequently, the majority of research conducted in understanding the clinical, pathological, and biochemical consequences of *LRRK2* mutation is limited to the mutations described above.

### LRRK2-associated phenotype

Given the high frequency of the p.G2019S mutation, the majority of *LRRK2* disease related clinical data are associated with this mutation. Most reports agree that the phenotypic features associated with *LRRK2* disease, characterized by unilateral tremor as initial symptom, good response to levodopa therapy, and slow, benign disease progression, closely resemble those seen in idiopathic Parkinson's disease (IPD). Tremor is the most commonly recognized initial symptom [7, 21]; indeed *LRRK2*-associated disease has been categorized by a large collaborative study as an asymmetrical tremor-predominant parkinsonism with bradykinesia and rigidity [22]. It has also been suggested that *LRRK2* associated disease may be marginally more benign than IPD. *LRRK2* mutation carriers usually present with lower risk of cognitive decline [22, 23] than IPD patients, and in general cognitive decline and psychiatric features are rarely reported in symptomatic *LRRK2* mutation carriers [22, 24]. Conversely a high frequency of depression, anxiety, and irritability, and a trend toward a greater risk of premorbid mood disorders have recently been reported in symptomatic *LRRK2* mutation carriers by two independent studies [25, 26]. Although dystonia and levodopa-induced dyskinesias (LID) seem to be more frequent in p.G2019S carriers than IPD, dystonia appears to be triggered by complications of medical or surgical treatments and the differences in LID did not reach statistical significances [22, 27].

There has been much discussion over the pentrance of *LRRK2* mutations, with estimates ranging from 30% to 80%. Initial estimates were likely skewed by the inherent ascertainment bias of family based studies, and by rather small numbers and age ranges for cohort based work. A more reasonable model has evolved over several years, being one that takes into account age with penetrance modeled in a large group of cases and controls from around the World. This shows that the penetrance of *LRRK2* mutations is clearly age dependent, increasing from 17% at age 50 to 85% at age 70 years [28]; notably some p.G2019S mutation carriers do not manifest disease even in their eighties or later [29].

Given the large number of asymptomatic p.G2019S mutation carriers, numerous reports are now focused on these healthy carriers in an effort to identify early preclinical biomarkers of PD. Within this context, lower cognitive performances have been reported in non-manifesting p.G2019S mutation carriers when compared to healthy non-carriers, suggesting cognitive impairment, though not commonly seen in symptomatic carriers, as a preclinical non-motor symptom of PD [30]. A higher frequency of postural and action tremor as well as gait alterations have also been reported in non-manifesting carriers [21, 31].

Olfactory dysfunction (hyposmia), a well-established non-motor feature of IPD that may precede disease onset and is present in 70–90% of patients with PD, has been widely studied in p.G2019S mutation carriers [32, 33]. Most reports agree that symptomatic p.G2019S mutation carriers also manifest olfactory dysfunction, although this is less frequent than in IPD [34, 35]. Similar findings have been reported in p.R1441G mutation carriers [36]. On the other hand, healthy mutation carriers exhibited similar levels of hyposmia to healthy non-carriers, including non-manifesting relatives, raising the possibility that the occurrence of this non-motor symptom of PD may be independent of the *LRRK2* mutation, or quite a late event in the disease process [21, 37].

In short, the *LRRK2*-associated phenotypic spectrum largely resembles the idiopathic disease and although some efforts have been carried out to establish pre-motor biomarkers for PD in *LRRK2* patients, more work remains to be done toward this end.

### LRRK2-associated neuropathology

Postmortem data available for about 40 *LRRK2* mutation carriers has revealed that the *LRRK2*-associated neuropathology is fairly heterogeneous. Although it is mainly characterized by the loss of dopaminergic neurons and the presence of Lewy bodies (LBs) and Lewy neurites (LNs), these are not present in all cases and the same mutation can cause quite diverse neuropathology [38, 39]. Despite this, the p.G2019S mutation is often associated with Lewy body pathology and neuronal loss in the substantia nigra (SN), tau pathology without LBs or LNs, and neuronal loss restricted to the SN, indicating that *LRRK2* mutation does not always manifest as synucleinopathy or LB disease [40]. Similar findings have been reported for the p.I2020T mutation, where five out of six mutation carriers exclusively showed tau pathology while tau-positive lesions, restricted to the brainstem along with alpha-synuclein deposits, were unique to one patient [41]; the p.Y1699C mutation, who carriers showed nigral neuronal loss and gliosis with either cortical and brainstem LBs or ubiquitin-positive cytoplasmic and nuclear inclusions [42, 43]; and the p.R1441C mutation, in which available data from four patients also revealed variable synuclein and tau pathology: LBs and LNs were detected in two cases (one with brainstem LB disease and one with diffuse LBdisease), neurofibrillary tangles (NFT) without either LBs or LNs were identified in a third patient, while neither the presence of LBs nor NFT were found in the fourth patient [44]. The only case examined with the p.R1441G mutation showed loss of dopaminergic neurons in SN without alpha-synuclein inclusions [45]. More recently, the p.N1437H mutation has also been associated with almost a high degree of dopaminergic cell loss in the SN pars compacta, brainstem, and cortex but sparse alpha-synuclein pathology, and pronounced ubiquitin-positive pathology in the brainstem, temporolimbic regions, and neocortex [46].

In conclusion, *LRRK2*-associated pathology, although mainly characterized by pure nigral neurodegeneration, is strikingly heterogeneous and can additionally present as tau-, alpha-synuclein-, TDP-43(one case), or ubiquitin-positive pathologies [39].

### LRRK2 mutation modifiers

The reduced penetrance and the variability in the age at onset (AAO) and neuropathology identified in *LRRK2* mutation carriers suggests that *LRRK2*-associated PD is probably modulated by a combination of both environmental and genetic factors. Considering the neuropathological findings in *LRRK2* mutation carriers, one might speculate that both *SNCA* and *MAPT* genes, which in turn are associated with the risk of PD [47], may also affect the *LRRK2*-related phenotypic expression. Indeed, two different studies have recently reported that two different *MAPT* alleles (rs2435207 and rs11079727) substantially affect the age at onset of motor symptoms in *LRRK2* mutation carriers. In both studies heterozygous carriers of the minor alleles (rs2435207-A and rs11079727-A) developed the parkinsonian symptoms approximately seven years later [48, 49]. Additionally, two *SNCA* polymorphisms (rs356165 and rs356219) have been identified as modifiers of the age at onset in both IPD and *LRRK2*-related PD by two independent studies, respectively. One study demonstrated that both heterozygous and homozygous IPD carriers of the major allele, rs356165-G, presented with earlier AAO than homozygous carriers of the minor allele (rs356165-A) with approximately 3 years of difference [50]. A second study showed that PD patients carrying the p.G2019S mutation also presented with earlier AAO when jointly carried the *SNCA* rs356219-G allele; in this study patients developed the parkinsonian symptoms approximately 9 years earlier than carriers of the homozygous rs356219-A allele [51].

Although these results were carried out in quite small cohorts they provide the first evidence supporting the notion that varied presentation of *LRRK2* disease may arise from a combination of multifactorial events. Further research is required to support these results and extend the search for modulating environmental and genetic factors that may affect the clinical presentation of *LRRK2* disease.

## Beyond Disease Causing Mutations: LRRK2 and Risk

As discussed above, the first PD linked variants identified in *LRRK2* were essentially disease causing mutations, each being relatively rare in the general population. What has become apparent over the last 9 years is that the genetic contribution of this locus to PD is not merely limited to disease causing mutations, but also includes variants that impart varying degrees of risk for this disease.

### Common protein-coding LRRK2 variants as risk factors for disease

In the wake of the original *LRRK2* mutation reports, a large number of screening efforts were performed both to define PD linked variants and to characterize the genetic variability in and around *LRRK2*. As described above, this work produced a large list of variants linked to disease, the majority only tenuously. Initial efforts failed to create a convincing argument that common risk variants existed at this locus [52–54], however, after several years of effort the relationship between common variants at this locus and risk for disease has become clearer.

**Fig. 2:**
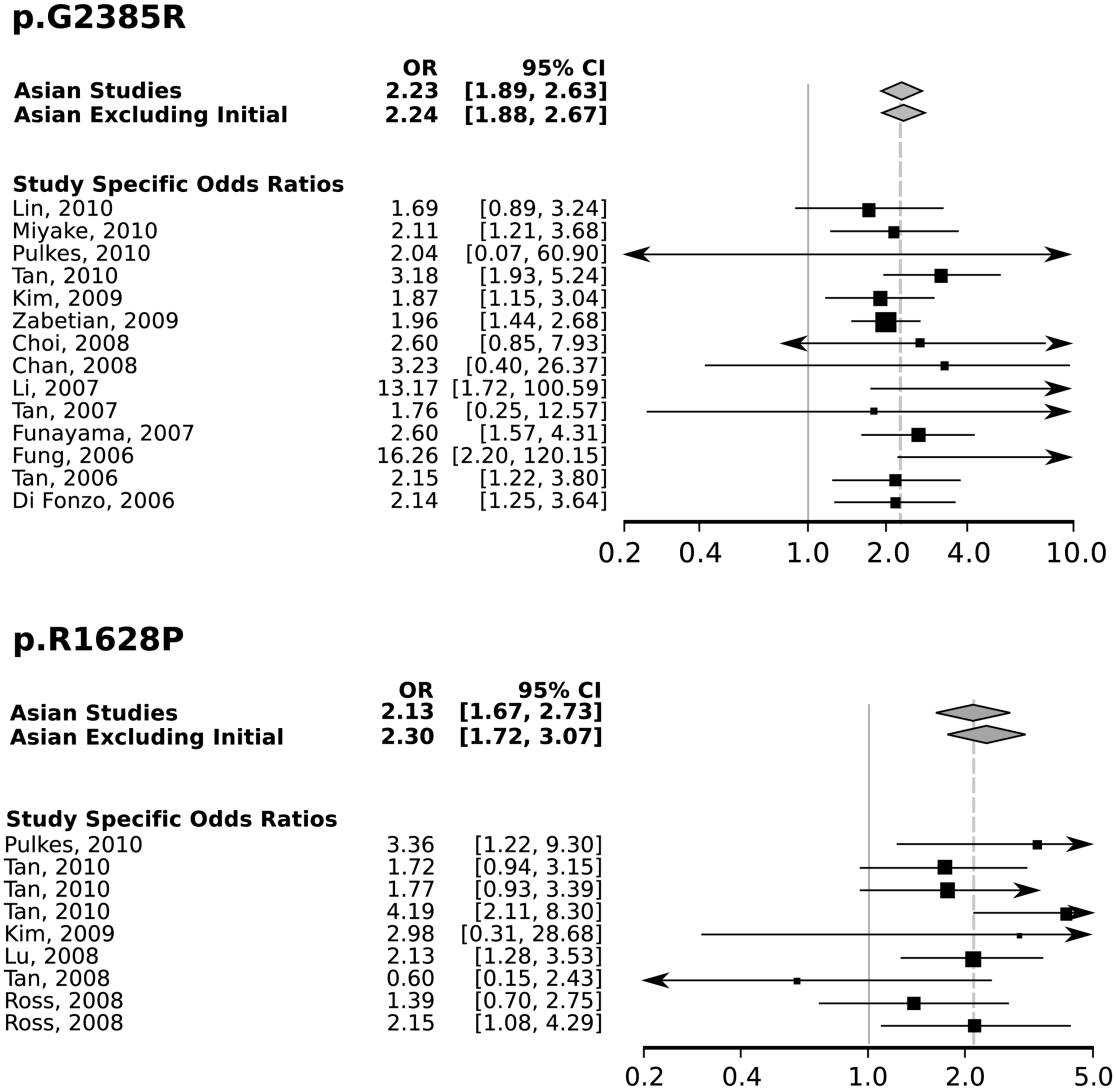
Forest plot showing risk associated with the p.G2385R Lrrk2 variant. Modified from PDGene (http://www.pdgene.org/meta.asp? geneID=13) [69] to show only studies performed in Asian populations. Data abstracted in January 2013.

As a part of a mutation screening effort, the LRRK2 variant p.G2385R was identified as a possible cause of PD in a father-daughter pair of patients originating from Taiwan [55]. Less than a year after this finding, it became apparent that this alteration was unlikely to cause PD, but may play a more interesting role in disease. In 2006 Vincenzo Bonifati led a study testing *LRRK2* variants in a case control population from Taiwan. This groups data showed p.G2385R to be relatively common in the control population, being carried by about 1 in every 20 people free from disease, certainly too common to be considered a mutation, but that this variant was twice as common in PD patients [56]. This work, which was performed in a series of ∼600 cases and ∼375 controls, therefore suggested that the p.G2385R increases risk for disease by about 2 fold; while this was only a relatively modest effect it has certainly been borne out by subsequent studies. Immediately after this initial result 2 studies confirmed this association both in Taiwanese and ethnic Chinese patients from Singapore [57, 58]. Since then a large number of reports have investigated this polymorphism and confirmed an association in varied Asian populations including Taiwanese, Singaporean Malays, Han Chinese, Hong Kong Chinese, Korean, and Japanese [59–68]. An excellent and frequently updated synopsis of these findings is maintained at the online PDgene repository (http://www.pdgene.org/meta.asp?geneID=13) [69], also adapted here (Fig. 2). While the magnitude and effect of p.G2385R has been borne out consistently in Asian populations, this alteration has essentially been too rare in other populations to observe any disease-linked effect.

A second coding variant, p.R1628P has been examined extensively across Asian populations following an initial report suggesting it conferred risk of a similar magnitude to p.G2385R [70]. In this original work Ross and colleagues identified p.R1628P as a risk factor in ethnic Chinese populations from Singapore and Taiwan, conferring an increased risk of 1.84 fold (95 CI 1.2–2.8). Similar to p.G2385R, the p.R1628P variant exhibits a low allele frequency, being present in ∼3% and ∼6% of ethnic Chinese controls and cases respectively. p.R1628P was described as rare or absent in Indian, and Japanese subjects. Examination of this variant in a series of Malay patients showed no association with disease, however, this likely reflects the insufficient power of this small series to detect an effect of the expected magnitude rather than a genuine lack of effect [70, 71]. Replication of the association between p.R1628P and disease has been largely consistent and has been performed in Han Chinese, and Korean patients [65, 72–75] (Fig. 2). Further an association with PD was identified in the Thai population, and given the high observed frequency of the variant allele in this population, the authors argued that this variant may have emerged from a Thai founder [76].

One of the most comprehensive studies into the role of *LRRK2* variants in disease was centered on genotyping of 121 exonic variants within the gene across a series of ∼7000 Caucasian patients, ∼5600 Caucasian controls, ∼1400 Asian patients, and ∼1000 Asian controls [17]. The authors gathered a series of rare and common variants from published studies, public databases, and their own sequencing data, then tested association for these across these large series of cases and controls. This work supported a role for p.G2385R as a risk variant in the Asian population, although not p.R1628P. In the Caucasian series the authors noted association between p.M1646T and disease (OR 1.43, 95% CI 1.15–1.78). This variant had been tested previously as a putative risk allele for PD with no obvious association, however, each of these small studies were relatively unlikely to yield positive results for a variant conferring such a modest effect size [77–79]. In the same study the authors also identified an association between protection against disease and a 3 variant haplotype p.N551K-p.R1398H-p.K1423K, the data in this study being consistent with a general protective effect in both Caucasian and Asian series [17]. These three variants exist in strong linkage disequilibrium, so it is not yet clear which of the three (or which combination) is the biological effector.

Lastly, in the work by Ross and colleagues p.A419V was posited as a potential risk variant in the Asian population [17]. While replication has been attempted, thus far the results remain inconclusive [80–83].

### Common non-coding LRRK2 variants as risk factors for disease

The majority of replicated genetic association loci for complex traits are not linked to obvious protein coding alterations and it is believed that these are likely to exert a biological effect through mediating expression and splicing [84]. It would appear feasible then that non-coding risk variants exist at the *LRRK2* locus in addition to protein coding risk variants. While initial efforts to show that this may be true were largely unsuccessful there has been increasingly compelling evidence of an association at *LRRK2* with disease from genome wide association studies. The first suggestion of this effect was seen by two coordinated studies in 2009 performed in Asian and Caucasian cohorts [47, 85]. This association in both the Caucasian and Asian data was most prominent 5′ to the protein-coding region of *LRRK2* and was independent of the well known p.G2019S mutation. The association has been replicated in subsequent well-powered association studies [86, 87]. These studies have shown quite modest effects of risk alleles at *LRRK2* with odds ratios in the range of ∼1.2-1.3, consistent with that seen at most other risk loci identified by GWA.

### Future work on LRRK2 and disease risk

As discussed there is good evidence that variability in and around *LRRK2* contributes to risk for PD and that *LRRK2* accounts for a greater proportion of the genetic architecture of PD than previously appreciated. The website PDGene goes some way toward identifying *LRRK2* variants that confer risk by compiling existing data; however, there is a need to assimilate current genetic data on *LRRK2* variants and perform more extensive data generation in an effort to unequivocally define alleles associated with risk for, and plausibly protection against, disease. Given the large size of this gene, direct genotyping, sequencing, and replication would have the benefit of providing information on linkage disequilibrium of variants, and establishing independency of effects. This type of work is not only important because it clarifies the role this gene product plays in disease, but also because it is key that scientists trying to understand the functional basis of lrrk2 in disease know which are truly associated variants, and which are not. From a genetic perspective this must be one of our research priorities in understanding *LRRK2* and disease.

There are a few as yet answered questions regarding the association of common non-coding risk variants near *LRRK2* and disease. Firstly, are there several non-coding risk variants at *LRRK2*; secondly, what is (are) the biologically relevant variant(s); and thirdly, what is the biological consequence of this variant. It is likely that fine mapping and resequencing efforts may go some way toward answering the first two questions. It is reasonable to assume that non-coding variants exert their effect through mediating expression of proximal transcripts, and good evidence exists to support this idea [88]; to date there is not compelling evidence of such an association for the *LRRK2* risk alleles. There are many reasons why this may be, the change in expression may be too subtle to detect with current methods, it may be only apparent under certain states or in certain cell types, or it may effect splicing or UTR usage rather than the overall level of transcript. It is likely that the increasing availability of reference data sets for genotype-expression-epigenetic correlation and improved methods for transcript profiling may help in dissecting out the relationship between risk alleles and biological consequence.

A clear trend in PD genetics is the existence of pleomorphic risk loci (PRL), i.e. individual genetic loci where several types of genetic risk for disease exist, including rare risk variants, common variants, and rare disease causing mutations [89]. As a general rule the genetics field has been most successful at identifying disease-causing mutations and common risk alleles. This latter category has been identified both by candidate gene association studies, which when performed in genes linked to monogenic forms of disease implicitly test the PRL hypothesis, and by genome wide association. There has been increasing interest in another area of the landscape of PRL, rare variants that exert moderate and minor risk. This category of risk alleles is often thought to be responsible for a major portion of the missing heritability of complex diseases such as PD. While there has been recent success in the identification of this type of risk variant in Alzheimer' disease [90, 91], the identification of such variants is challenging. This work usually requires very large sample series, and, particularly in the context of very rare variants, an ability to collapse groups of genetic variability into potential risk and potential protective prior to association testing. While this type of categorization is often performed computationally, based on a prediction of consequence (specifically protein coding consequence), there is the potential to incorporate expression and splicing consequences of rare variants into such a model. As with fine mapping of loci, it is likely that resequencing, in large series, will aid in the identification of rare risk alleles at the *LRRK2* locus.

## The Function of LRRK2 and its Role in Disease

As we have learned more about the genetics of *LRRK2*, the importance of this gene in Parkinson's has become ever clearer. It is a large leap, however, from human disease genetics to therapeutic targeting and one that is dependent upon a clear understanding of both protein structure and function. What, then, have we learned about the function of LRRK2 since mutations in the gene were first described? LRRK2 is a large multidomain enzyme, coupling kinase and GTPase activities with a number of protein/protein interaction domains [92]. It is one of a small number of proteins in the human genome that possess more than one enzymatic activity in the same open reading frame, an aspect of LRRK2s biology that is both attractive (as it presents two active sites to target) and complicates interpretation of both its normal cellular role and its dysfunction in the disease state. Over the last ten years a huge amount has been learned about the biochemistry and biology of LRRK2, and although a clearer picture is beginning to emerge regarding the consequences of mutations on the function of this protein there are still large gaps in our understanding of the pathogenic pathways that link LRRK2 to Parkinson's disease.

### Structural biology

The complete open reading frame of LRRK2 codes for a 2527 amino acid protein, and initial domain prediction analysis of the primary sequence of LRRK2 identified two enzymatic domains: a GTPase and a kinase [93]. The GTPase domain was categorised as a Ras of Complex Proteins, or ROC, domain while the kinase domain has been variously described as belonging to the MAPKKK family, to the Mixed Lineage Kinases or to the RIP kinases [94]. Based purely upon the primary sequence of the kinase domain, LRRK2 is most closely linked to the RIP kinases. The two enzymatic domains of LRRK2 are separated by a domain of unknown function, a c-terminal of ROC (COR) domain, and are flanked by a number of protein/protein interaction motifs including a WD40 domain, the Leucine Rich Repeats that give the protein its name, and a series of repeats in the N-terminal region that have described as ankaryin repeats and armadillo repeats (Fig. 1) [95].

A number of atomic resolution structures have been reported for fragments of LRRK2 or closely related members of the same protein family. The first to be described was a fragment of human LRRK2 coding for the ROC domain of the protein, revealing a domain swap dimer structure [96]. Subsequent to this, a crystal structure has been elucidated for a prokaryotic homolog of LRRK2 isolated from Chlorobium tepidum, supporting a dimeric structure for this ROCO protein [97]. The chlorobium tepidum protein differs from LRRK2 in that it lacks a c-terminal kinase domain, but provides the first structural detail for the enigmatic COR domain – suggesting that this domain provides the key surfaces for dimeric interaction. Finally, the kinase domain of a LRRK2 ortholog from Dictyostelium has been crystalized and a 1.8 *Å* structure derived [98]. It is noteworthy that the publically available structures for LRRK2 highlight the extent of the challenge that LRRK2 presents to structural biologists: thus far, only small fragments or homologs of LRRK2 have been purified to the point where structural studies are possible.

Based upon the published structures for LRRK2 and related proteins, Gasper and co-workers have proposed a model for ROCO protein function based upon Guanosine nucleotide dependent dimerization (GAD), classing LRRK2 as such a protein [99].

Other than purely structural approaches, a number of studies have been carried out to investigate the complex formed by LRRK2 under cellular or *in vitro* conditions. Using techniques such as size exclusion chromatography and blue native gel electrophoresis, several groups have reported that LRRK2 forms a complex consistent with a dimeric conformation [100–102], although there are reports in the literature that conflict with this [103]. Further studies using electron microscopy also support a dimeric conformation [104], and taking all of the extant data into consideration it is likely that dimerization plays an important part in the function of LRRK2 in a cellular context. A recent study using total internal reflection microscopy suggests that LRRK2 is predominantly monomeric in the cytoplasm, and forms a multimeric complex when associated with membranes [105]. The elucidation of the precise atomic structure of this complex, and the dynamics of this interaction in a functional context, remain a huge challenge.

### Enzymatic function

The presence of two predicted enzymatic domains in the LRRK2 open reading frame has focused a considerable research effort on characterising and defining these activities. Soon after the description of mutations in LRRK2 linked to PD, a series of papers reported that LRRK2 was indeed an active kinase [106–108]. Similarly, several groups have reported that LRRK2 possesses a functional GTPase activity [109–111]. One of the many puzzling aspects of LRRK2 biology is how these two enzymatic activities relate to one another. Based upon a study investigating GTPase and kinase function in LRRK1, a close human homolog of LRRK2, which revealed that the kinase function of this protein was dependent upon GTP binding by the ROC domain [112], a number of publications have examined whether the kinase activity of LRRK2 has a similar requirement [96, 113–115]. This led to the development of a model for LRRK2 enzymatic activity analogous to that applied to the small GTPases such as Ras, where the tightly controlled cycle between GTP and GDP bound states governs the activity of interacting kinases (in the case of Ras, the kinase Raf). More recent studies have suggested that the kinase activity of LRRK2 is dependent not upon whether GTP or GDP occupies the active site of the ROC domain, but upon whether there is a Guanosine nucleotide of any description within the active site [116]. Although by no means proving that the GAD model for LRRK2 function is correct, these data are certainly consistent with a Guanosine nucleotide dependent activation of kinase activity.

Intriguingly, there is accumulating evidence that the kinase activity of LRRK2 can be directed against its own ROC domain [117, 118], suggesting that there is a complex, reciprocal relationship between the enzymatic activities of this protein. Even more intriguing is a report that phosphorylation of the ROC domain can have a functional impact on the properties of this domain [119]. It should be noted that much of the data relating to phosphorylation of the ROC domain of LRRK2 derives from *in vitro* model systems, and this has not yet been demonstrated to be a physiologically relevant phenomenon, therefore caution should be exercised in interpreting these results.

Outside of the ROC/COR/Kinase enzymatic core of LRRK2, it has become clear that the far c-terminus of LRRK2 plays a crucial role in regulating the kinase activity of this protein. Loss of the WD40 domain ablates the kinase activity of LRRK2, and provocatively even the loss of the seven amino acids at the far c-terminus of LRRK2 (residues 2520–2527) results in a kinase inactive form of the protein [120, 121]. How the WD40 domain can have such an impressive impact on kinase activity is not clear, and the impact of c-terminal truncation on LRRK2s GTPase activity has not been thoroughly investigated. It is likely that the underlying mechanism relating the WD40 domain to the enzymatic activities of LRRK2 will not be completely clarified until detailed structural information describing the precise spatial relationships of the different domains of LRRK2 is elucidated.

One final aspect of the control of LRRK2 enzymatic activity, linking in with the GAD model for ROCO protein function, is the dependence of kinase activity in particular upon dimerization. Although it is technically challenging to determine whether this kinase activity is derived from one species of LRRK2 rather than another (dimeric versus monomeric), there is evidence from several experimental conditions that the kinase activity of LRRK2 is dependent upon dimerization [100, 101, 122]. Clearly, more work is needed to determine if dimerization is an absolute requirement for kinase activity or if multiple LRRK2 complexes are enzymatically active [123].

### Signaling pathways and cellular function

The cellular function/functions of LRRK2 have been a matter of great debate ever since the initial report of mutations in LRRK2. Given its large size and complex domain structure, it is perhaps not surprising that LRRK2 has been implicated in a startling array of cellular tasks.

The starting point for many of these roles has been LRRK2s status as a kinase, GTPase and putative signalling scaffold [92, 124]. A number of studies have placed LRRK2 in one of the many established signalling cascades, including the mTOR, ERK, WNT and TLR pathways (Figure) [125–128]. The key challenge for these studies has been the validation of LRRK2s involvement by the demonstration of a physiological LRRK2 dependent phosphorylation event, and, despite almost ten years of searching, a clear validated substrate for LRRK2 has yet to emerge.

One aspect of LRRK2 biology that has stood the test of repeated investigation is an interaction with 14-3-3 proteins, dependent upon phosphorylation at residues S910 and S935 [129–131]. Phosphorylation and binding of 14-3-3 proteins plays an important role in the cellular localisation of LRRK2, although the kinase(s) responsible for directly phosphorylating LRRK2 at these residues has not been identified. Indeed, studies of the cellular localisation of LRRK2 have provided some clues as to what it may be doing within the cell. Data from a number of groups, using a variety of approaches, suggests that LRRK2 can associate with membrane structures in the cell [132–134]. Biochemical studies and total internal reflection microscopy have provided evidence that LRRK2 can cycle between a cytosolic, monomeric form which has decreased activity, and a membrane associated, multimeric form that has a higher activity [105, 122].

**Fig. 3:**
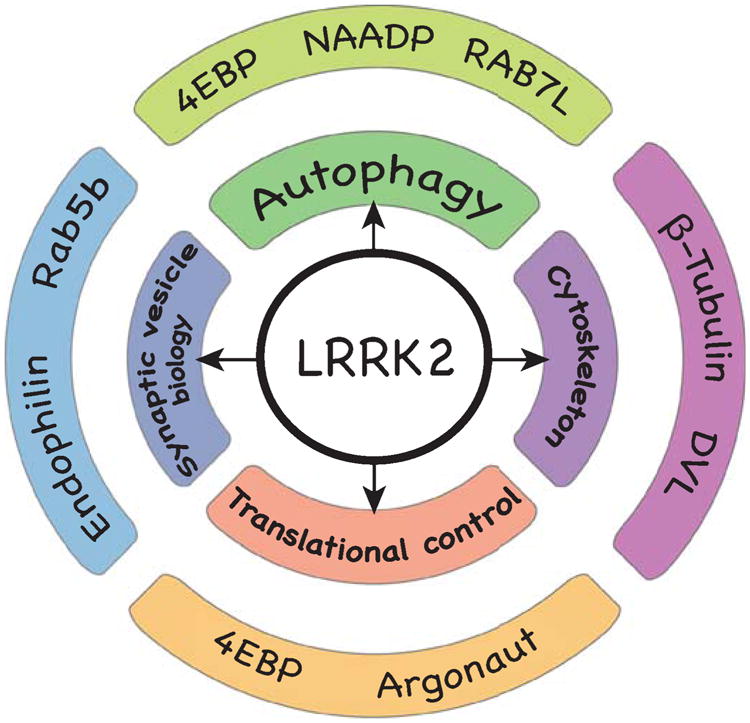
The biology of LRRK2. LRRK2 has been implicated in a host of different cellular processes.

With regard to the physiological processes that these cascades link LRRK2 to, the recurring cellular themes are vesicle cycling, autophagy, miRNA processing and cytoskeletal regulation (Fig. 3). It is not yet clear if these represent independent or convergent roles for LRRK2, and it is certainly not beyond the bounds of possibility that LRRK2 might have important roles in a number of cellular processes given its complicated domain structure [123].

A number of studies have highlighted a role for LRRK2 in the regulation of synaptic vesicles [135, 136], with Matta and coworkers demonstrating that LRRK2 interacts directly with endophilins to exert an influence on vesicle endocytosis. Further evidence of a role for LRRK2 in vesicular biology comes from studies linking LRRK2 to cellular macroautophagy [128, 137–139], although it is not clear precisely how LRRK2 impacts on this process. A strong line of evidence supporting a role for LRRK2inautophagy, albeit a complicated role, comes from studies of mice lacking LRRK2 [140, 141]. In these mice, knockout of LRRK2 results in a kidney phenotype marked by inclusions and by alterations in markers for autophagy – although again the precise reason why this occurs is not clear. The complications arise from an apparent biphasic impact on autophagy, with autophagic markers altering through development. A more recent study has implicated LRRK2 in chaperone-mediated autophagy (CMA), suggesting that alterations in LRRK2 can result in defective CMA [142]. Macleod and coworkers have reported LRRK2 interacting with Rab7L1 to influence protein sorting, including lysosomal protein sorting, via the retromer complex – suggesting that multiple systems surrounding protein disposal can be adversely impacted by LRRK2 [143].

As noted above, miRNA regulation is another recurring theme in LRRK2 biology. Gehrke and colleagues described LRRK2 regulating let-7 and miR-184* to control protein translation, with a subsequent study by Cho et al reporting LRRK2 levels being regulated in turn by miR-205 [144, 145]. How LRRK2 regulates, and is regulated by, miRNAs is an area of active investigation. It will be of particular interest to discover if these interactions have a consistent pathological role in human cell models and brain tissue.

LRRK2 has been identified as interacting with a number of cytoskeletal proteins, including β Tubulin [146, 147], actin [148] and Moesin [120]. There is increasing evidence linking LRRK2 to a functional role in the control of cytoskeletal remodeling, which is of particular interest given that one of the more robust cellular phenotypes associated with LRRK2, alterations in neurite branching, can be impacted by this [149, 150].

One area of LRRK2 biology that is under increasing scrutiny is a putative role in the immune system. It has long been noted that LRRK2 is highly expressed in immune cells, and several studies have shown that LRRK2 is involved in the response to pathogens and interferon γ [151, 152]. Intriguingly, the LRRK2 locus been implicated in Crohn's disease and susceptibility to leprosy, providing a genetic link to immune disease [153, 154] and recent studies have highlighted a potential role in microglial response within the brain [155, 156]. How these data tie in to the cellular biology of LRRK2 is unclear, but this is certainly an area that merits further investigation.

### Mutations in LRRK2 and their impact on biology

The central question in LRRK2 biology, and the question that drives the majority of research into this protein, is straightforward: how do the mutations in LRRK2 linked to PD result in disease? The answer, unfortunately, is convoluted. From a genetic standpoint, as highlighted earlier in this article, it is clear that the enzymatic core of LRRK2 is central to the role of this protein in neurodegeneration as the penetrant mutations unambiguously linked to disease all cluster within the ROC/COR/kinase triptych of domains [17]. To date, however, no consistent biochemical impact of these mutations has been reported (Table 1). Mutations in the ROC and COR domains (for example those at codon 1441 and the p.Y1699C mutation) act to decrease GTPase activity by an as yet undefined mechanism, although based on the GAD model for LRRK2 function one could hypothesise a disruptive influence on dimer formation for both of these residues. A detailed characterisation of the impact of mutations in the kinase domain (either the p.G2019S or p.I2020T mutants) upon GTPase activity of full length LRRK2 has not yet been carried out, however the p.G2019S mutation results in a 20% reduction in GTPase activity compared to wild type LRRK2 in assays using a truncated recombinant form of the protein [157]. This suggests that the GTPase activity of LRRK2 is certainly worthy of further investigation. The impact of mutations throughout the ROC/COR/kinase domains upon kinase activity has been assessed by a number of groups, with a substantial and consistent impact noted for p.G2019S but not other mutations (including the p.I2020T mutation, located at the residue next to p.G2019) [158]. In a surprising twist, a recent study has suggested that a risk variant in the WD40 domain of LRRK2 found associated with PD in asian populations (the p.G2385R polymorphism) substantially decreases the kinase activity of LRRK2 – the opposite of the impact of the p.G2019S mutation [121]. If correct, this result has profound implications for the development of inhibitors targeting the kinase activity of LRRK2 as a therapeutic avenue. An example of the complexities of enzymatic activities and inhibition in a cellular context is presented by a recent analysis of Wnt signaling linked to LRRK2 – with both mutations (including the p.G2019S mutation, and therefore a hyperactive kinase) and kinase inhibition resulting in the same cellular alteration in Wnt signaling [124]. A comprehensive analysis of the impact of mutations on the folding and turnover of LRRK2 has not been reported, however there are suggestions that the p.R1441C and p.I2020T mutations could alter protein stability and/or protein turnover – which would, of course, have a significant impact on enzymatic activity [159, 160]. Despite almost ten years of research, we are still unsure as to what aspect of LRRK2s biochemistry we should be concentrating on in the context of Parkinson's disease. If any theme emerges looking across the extant literature covering the biochemical impact of mutations in LRRK2, it is that disruption of enzymatic activity – whether up or down, kinase or GTPase – can be deleterious. This suggests a model where LRRK2 activity is tightly regulated within cells, and it is the disruption of this regulation regardless of the direction that results in the instigation of disease.

Unfortunately the picture is just as murky when the impact of LRRK2 mutations on cellular phenotypes is considered. One of the few consistent aspects of LRRK2 mutations in a cellular context is that they display increased toxicity compared to the wild type protein, and this toxicity is dependent upon kinase activity [108, 115]. What drives this toxicity is not known, although there is no shortage of possibilities - ranging from FADD signalling through to changes in translational regulation [144, 161]. A major stumbling block, similar to that encountered when assessing the biochemical impact of mutations in LRRK2, is that different mutations quite often have different outcomes with regard to a given cellular readout. A good example of this is provided by the robust interaction between LRRK2 and 14-3-3 [129, 130]. While the p.R1441G, p.Y1699C and p.I2020T mutants all have a major impact on the phosphorylation of LRRK2 at p.S910 and p.S935 (and thus a decreased binding of LRRK2 to 14-3-3), the common p.G2019S mutant does not. Conversely, a number of cellular phenotypes linked to PD mutations in LRRK2 – for example the impact on miRNA processing described by Gehrke and coworkers - alter in the presence of the p.G2019S mutation, but not other mutations. This poses a particular conundrum to researchers: we know that kinase activity is important for LRRK2 and its pathogenic impact, and we know that the p.G2019S mutation that alters kinase activity is the most common mutation in LRRK2, but it is equally clear that kinase activity, or at least a simple increase in this activity, is not the only factor in the pathogenic process. The challenge, therefore, is to distinguish between phenotypes that are primarily driven by the kinase activity of LRRK2 (particularly the case with the p.G2019S mutation) and those that are truly associated with disease. One obvious way to address this is to examine mutations in each of the domains of LRRK2, for example the p.R1441G, p.Y1699C and p.G2019S substitutions (with the possible addition of the p.G2385R polymorphism in the WD40 domain), for any phenotype that is suspected to link with LRRK2. It is of course conceivable that different mutations in LRRK2 have different mechanisms of action, but, given the consistent clinical phenotype associated with LRRK2 mutations, it is highly likely that there is a property that unites all of the mutations in LRRK2 – the problem is that we haven't found it yet.

### Animal models for LRRK2 dysfunction

Given the interest in LRRK2 and its role in PD, it is perhaps not surprising that a large number of animal models for LRRK2 dysfunction have been developed. Drosophila melanogaster, Danio rerio and Caenorhabditis elegans models have been developed, including knockout, knockdown and overexpression of the human protein (plus and minus mutations) or the equivalent fly, fish or worm orthologs [125, 162–168]. The interpretation of the data from these models is complicated by the relationship between human LRRK2 and the LRRK genes found in these species: in all three cases there is only one LRRK gene, and whether this corresponds to human LRRK1 or LRRK2 is a matter of some debate [169].

Rodents, in contrast, have orthologs of both human LRRK genes and a number of mouse and rat models for LRRK2 have been developed over the last nine years (summarised in reference [170]). In common with rodent models for other genes linked to Parkinson's disease, these have proved to be somewhat disappointing in terms of replicating a phenotype that is equivalent to the human disorder [171]. Important insights into LRRK2 dysfunction have been gained, however, by detailed analysis of these models. A putative role for LRRK2 in modulating alpha synuclein pathology was reported by Lin and co-workers examining transgenic mice expressing LRRK2 and the p.A53T form of alpha-synuclein, and several models using both BAC expression of LRRK2 mutants and viral mediated acute expression have reported accumulation of tau [172–175]. These findings are particularly interesting in the light of the varied pathology observed in human LRRK2 mutation carriers. Echoing the vesicular data from cellular models, the p.R1441C mutation impacts on dopamine cycling in mice [176]. The extant transgenic models for LRRK2 do not, on the whole, present with neuronal degeneration, however acute expression of LRRK2 via viral transduction provides evidence of direct neuronal toxicity in an *in vivo* setting [177, 178]. How this acute expression relates to the drawn out pathogenic process seen in human PD is unclear. Knock out rodent models for LRRK2 have likewise revealed important insights into LRRK2 biology – providing evidence of a link to autophagy and to Crohn's disease [140, 179].

### Future functional challenges

As is abundantly obvious from this brief summary of our current understanding of LRRK2 biology, there are many challenges still to be met before we have a complete picture of LRRK2s function and how this is perverted in Parkinson's disease. Two even larger challenges are on the horizon. First, and dependent upon elucidation of the role of mutations in LRRK2 in the pathological cascade that leads to Parkinson's disease, will be how to correct their impact. Based upon the indubitable importance of kinase activity in the aetiopathogenesis of LRRK2 linked PD, there is a concerted effort underway to develop inhibitors of LRRK2 kinase activity [180]. It is by no means clear, however, if this approach will succeed in a clinical setting–although it is likely that LRRK2 kinase inhibitors will be tested in a clinical trial sooner rather than later.

The second major challenge is to dissect the role of *LRRK2* in sporadic PD. The scale of this challenge on a cellular scale is difficult to underestimate. We have, to date, struggled to decipher what mutations in *LRRK2* strongly associated with disease do to the brain. How much more difficult will it be to understand how subtle variation at the *LRRK2* locus, accounting for a fraction of a percentage point of increased life time risk for PD, impacts of the pathogenesis of this disorder? At present it is not known what the association uncovered by the GWA studies at the *LRRK2* locus represents, and indeed uncovering exactly what the association drives at a functional level may be no small feat in itself.

### Summary

The field has made enormous progress since the discovery of *LRRK2* mutations in 2004. Our understanding of the genetic basis of this disease has increased exponentially since this time, and it is clear that varied forms of *LRRK2* exert many different types of influence on the disease process. Likewise, while there is still a long way to go in understanding the pathobiological basis of PD, *LRRK2* mutations have provided valuable insights into this process, and the tools with which to understand more. We believe that this is an exciting time in PD research, and we believe that LRRK2 will be central to this effort for a long time to come.

## Figures and Tables

**Table 1:** Summary of the impact of mutations in LRRK2 on biochemical readouts for LRRK2 function

Mutation	Domain	Enzymatic impact	14-3-3 binding
p.N1437H	ROC	Unknown	Unknown
p.R1441C	ROC	GTPase↓	↓
p.R1441G	ROC	GTPase↓	↓
p.Y1699C	COR	GTPase↓	↓
p.S1761R	COR	Unknown	Unknown
p.I2012T	Kinase	None	↓
p.G2019S	Kinase	Kinase ↑ GTPase↓	No change
p.I2020T	Kinase	None	↓
p.G2385R	WD40	Kinase↓	No change
